# Allostery‐driven changes in dynamics regulate the activation of bacterial copper transcription factor

**DOI:** 10.1002/pro.4309

**Published:** 2022-04-12

**Authors:** Idan Yakobov, Alysia Mandato, Lukas Hofmann, Kevin Singewald, Yulia Shenberger, Lada Gevorkyan‐Airapetov, Sunil Saxena, Sharon Ruthstein

**Affiliations:** ^1^ Department of Chemistry and the Institute of Nanotechnology & Advanced Materials, Faculty of exact sciences Bar Ilan University Ramat‐Gan Israel; ^2^ Department of Chemistry University of Pittsburgh Pittsburgh Pennsylvania USA

**Keywords:** copper homeostasis, CueR, EPR, metalloregulator, protein–DNA interaction

## Abstract

Metalloregulators bind and respond to metal ions by regulating the transcription of metal homeostasis genes. Copper efflux regulator (CueR) is a copper‐responsive metalloregulator that is found in numerous Gram‐negative bacteria. Upon Cu(I) coordination, CueR initiates transcription by bending the bound DNA promoter regions facilitating interaction with RNA polymerase. The structure of *Escherichia coli* CueR in presence of DNA and metal ion has been reported using X‐ray crystallography and cryo‐EM, providing information about the mechanism of action. However, the specific role of copper in controlling this transcription mechanism remains elusive. Herein, we use room temperature electron paramagnetic resonance (EPR) experiments to follow allosterically driven dynamical changes in *E. coli* CueR induced by Cu(I) binding. We suggest that more than one Cu(I) ion binds per CueR monomer, leading to changes in site‐specific dynamics at the Cu(I) binding domain and at the distant DNA binding site. Interestingly, Cu(I) binding leads to an increase in dynamics about 27 Å away at the DNA binding domain. These changes in the dynamics of the DNA binding domain are important for exact coordination with the DNA. Thus, Cu(I) binding is critical to initiate a series of conformational changes that regulate and initiate gene transcription.

**Broad audience statement:**

The dynamics of metal transcription factors as a function of metal and DNA binding are complex. In this study, we use EPR spectroscopy to measure dynamical changes of *Escherichia coli* CueR as a function of copper and DNA binding. We show that copper controls the activation of the transcription processes by initiation a series of dynamical changes over the protein.

## 1 INTRODUCTION

Bacteria are skilled survivors capable of living in environments with high concentrations of different metal ions.^1^ Some of these metals are essential, but even essential metals can become detrimental above a certain concentration within the cytosol. Therefore, bacteria have evolved as highly sophisticated and complex biological processes to regulate and maintain intracellular metal homeostasis.^2^, ^3^ One of these processes is controlled by metalloregulators, which respond to metal ions and regulate the transcription of genes that protect the bacteria from metal‐induced stress.^4^, ^5^

**FIGURE 1:**
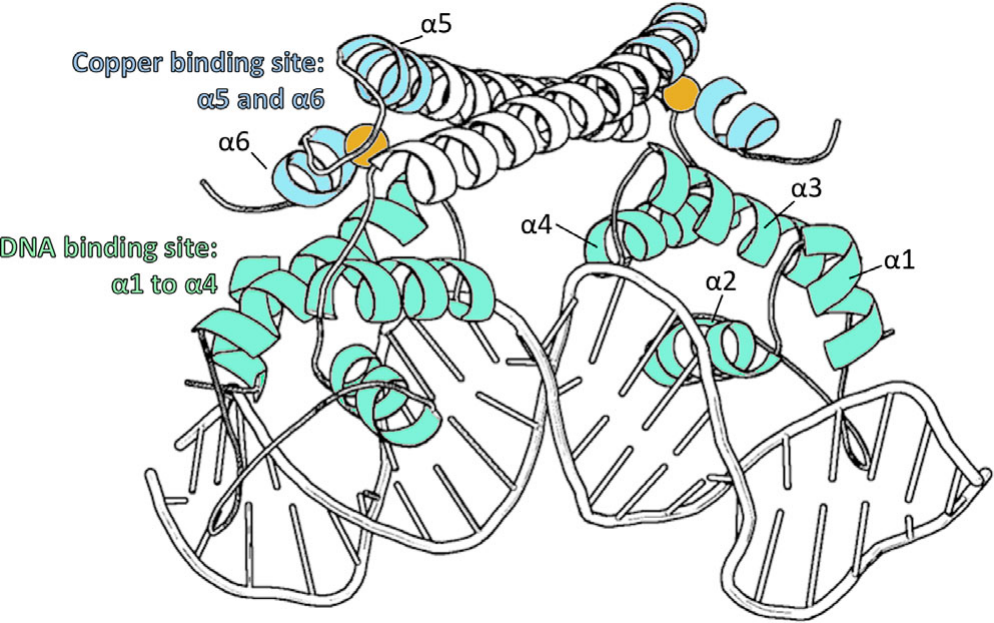
Schematic representation of holo‐CueR dimer bound to DNA (PDB: 4wlw). DNA binding site (α1 to α4) is colored in cyan while the copper binding site (α5 and α6) is depicted in light blue. Copper is colored according to the element and represented as a sphere

CueR, a representative member of the mercury resistance regulator (MerR) family of metalloregulators, controls expression of genes involved in copper homeostasis of bacteria.^6^, ^7^, ^8^ The mechanism of transcription initiation by CueR and other MerR family regulators is described by bending the spacer domain of promoter DNA, enabling the RNA polymerase complex to bind and subsequently initiate gene transcription. The coordination of Cu(I) to CueR facilitates DNA binding and activates transcription of copA and cueO genes.^9^, ^10^, ^11^ The X‐ray structure of *Escherichia coli* CueR in holo (in presence of Cu(I)) form was resolved,^6^, ^12^ showing that the CueR protein is a dimer, and each monomer has an ααββαααα secondary structure (Figure 1). The first four helixes, labeled α1 to α4, comprise the N‐terminal domain which binds and interacts with the DNA (Figure 1). The turn between α5 and α6 forms the high‐affinity Cu(I) binding site by linear Cu(I) dithiolate coordination, which involves two cysteine residues, C112 and C120 (Figure 1). Both apo‐CueR and holo‐CueR can coordinate to the specific DNA promoter. However, the presence of Cu(I) is critical in order to facilitate bending of the DNA.^11^ In this bent state, RNA polymerase can interact with the CueR‐DNA complex and initiate the transcription process.^7^, ^13^

Single‐molecule FRET (smFRET) measurements have shown that in solution, CueR can exist in four different states: apo‐CueR, holo‐CueR, apo‐CueR bound to DNA, and holo‐CueR bound to DNA.^14^ Initiation of transcription occurs when holo‐CueR is bound to the DNA, and termination occurs when holo‐CueR bound to the DNA is replaced by apo‐CueR.^14^, ^15^ Intriguingly, these smFRET measurements have also shown that two complexes of Cu(I)‐CueR‐DNA–RNA polymerase (RNAP) can be formed, where only one initiates transcription while the other does not. The authors called the latter a “dead‐end” complex.^16^ The role of DNA structure in transcription regulation has also been revealed by recent crystal structures of a CueR‐Ag(I)‐DNA complex and a complex of a C112S‐C120S mutant of CueR with DNA. The C112S‐C120S mutant lacks the metal‐binding site. Thus, the C112S‐C120S CueR‐DNA complex was deemed to represent the repressed state. It was shown that the largest difference between these two complexes was found in the DNA conformation.^12^ However, the DNA conformation was only examined at sub‐stochiometric ratio of protein to DNA in the apo‐protein–DNA complex. More recently, we have exploited DNA Cu(II) spin‐labeling to systematically measure the DNA conformations at various stages of the transcription cycle.^11^ Our results showed that apo‐CueR can bend the DNA but a high protein concentration is needed (in a ratio of 6 CueR:DNA). On the other hand, such bending is facile in the presence of Cu(I). These results suggest a clear structural picture of how apo‐CueR can replace holo‐CueR in the protein–DNA complex to terminate transcription. In general, these results show the value of systematic concentration‐dependent measurements to incisively identify the relationship of structure to function. Bending of the DNA in presence of Cu(I) and bound RNAP was also demonstrated by a recent cryo‐EM structure.^13^

The crystallographic data report changes in the DNA binding domain of the protein (Figure 1) between the repressed‐ and active‐state of CueR.^12^ We have also applied electron paramagnetic resonance (EPR) distance measurements to follow conformational changes that CueR undergoes as a function of Cu(I) and DNA binding in solution.^17^, ^18^ We revealed major changes in the structure, specifically between the apo‐CueR‐DNA and holo‐CueR‐DNA (with Cu(I)) complexes. In the holo‐CueR‐DNA complex (i.e., the active state), we were able to detect at least two different conformations of CueR in presence of Cu(I) and DNA. These experiments also showed that Cu(I) drives these conformational changes, in presence of DNA. Moreover, structural rearrangements in the DNA binding domain were previously reported by a different transcription regulator, the catabolite gene activator protein (CAP) that regulates transcription upon cAMP binding.^19^ These results motivated the present work which seeks to understand the role of Cu(I) in driving these conformational changes. In this work, we follow the dynamical changes that CueR undergoes at room temperature (RT) as a function of Cu(I) and DNA binding using room temperature (RT) continuous‐wave (CW) EPR experiments.

## 2 MATERIALS AND METHODS

### 2.1 CueR cloning and expression

The CueR sequence was isolated by PCR using *E. coli* genomic DNA with primers specific to the CueR N‐terminal (5’‐GCAGCGGCCTGGTGCCGCGCGGCAGCATGAACATCAGCGATGTAGCAAAAATTACCGGCC‐3′) and C‐terminal (3’‐GTCCACCAGTCATGCTAGCCATATGTCACCCTGCCCGATGATGACAGCAGC‐5′). These primers also contain flanking sequences of the pET28a expression vector. The same procedure was used to generate the different mutations, using specific primers containing the desired mutation. Briefly, site‐directed mutagenesis was performed by polymerase chain reaction (PCR) amplifcation using two primers, each containing a target‐specific sequence and a 5′ extension (non‐over lapping) that provide the suitable DNA bases for the desired mutation. The C129 and C130 cysteines do not affect DNA binding or CueR activity but they are accessible for spin labels. Therefore, these two cysteine residues were mutated into alanine residues. These constructs were then transfected to DH5α cells to amplify the plasmid. Next, the sequence was confirmed by sequencing and the purified plasmid was transfected to BL21 cells. Subsequently, the CueR constructs were expressed in BL21 cells, which were grown to an optical density of OD600 = 0.8 and induced with 0.5 mM isopropyl‐βD‐thiogalactopyranoside (CALBIOCHEM) overnight at 22°C. The cells were harvested by centrifugation at 8,000 x g for 20 min. Pellets were resuspended in 1:10 (w/v) 50 mM HEPES pH = 7.0, 0.5 M NaCl, 10% glycerol and 1 mM DTT. 2 mM of PMSF protease inhibitor (Sigma) was added to the cells. The cells were lysed by using a microfluidizer. The cell lysate was then centrifuged at 4°C for 30 min at 20,000× g. Next, the protein was purified from soluble fraction by Ni‐NTA agarose beads (Thermo Fisher Scientific), according to the manufacturer's protocol using 50 mM HEPES pH = 7.0, 0.5 M NaCl, 10% glycerol and 1 mM DTT including 20 mM imidazole as the loading and wash buffer. 10 CV of wash buffer was used to remove residual impurities. CueR was eluted by addition of 250 mM imidazole to the lysis buffer. The obtained fractions were dialyzed overnight. The dialysis buffer comprised of 50 mM HEPES pH = 7.0, 0.5 M NaCl, 10% glycerol, and 1 mM DTT. CueR purity was confirmed by 12% tricine SDS‐PAGE and Coomassie blue staining.

### 2.2 Electrophoresis mobility shift assay by fluorescence

The electrophoresis mobility shift assay by fluorescence (EMSA) experiments was carried out according to the published protocol.^20^ Briefly, 5% (wt/vol) non‐denaturing gels were poured and stored at 4°C until usage. The double‐stranded copA promoter DNA (5′‐TTGACCTTCCCCTTGCTGGAAGGTTTA‐3′) and CueR protein were incubated at room temperature for 30 min. Glycerol was added to the sample reaching a final concentration of 15% (v/v) prior to loading. Then, the gel was run with TAE buffer (40 mM Tris, 20 mM acetic acid, 1 mM EDTA) at 4°C and 80 V for 1 hr. Subsequently, the gel was stained with 1 μg/mL ethidium bromide in TAE running buffer for 30 min. The gel was then washed with TAE running buffer for 30 min. The stained gel was analyzed with a Gel Doc EZ BioRad System using the EtBr protocol.

### 2.3 DNA for EPR measurements

The same DNA promoter sequence containing 27 base pairs was used for the EPR measurements. This DNA fragment was isolated from the copA promoter, and the sequence includes a specific region known to bind CueR: 5′‐TTGACCTTCCCCTTGCTGGAAGGTTTA‐3′.^21^

### 2.4 CueR spin‐labeling protocol

The protein was labeled with S‐(2,2,5,5‐tetramethyl‐2,5‐dihydro‐1H‐pyrrol‐3‐methyl) methanethiosulfonothiate (MTSSL, TRC) at the targeted cysteines. CueR was initially incubated with 10 mM DTT and Cu(I) ([CueR‐monomer]:[Cu(I)] = 1:20) overnight. DTT was removed by dialysis using ADELAB SCIENTIFIC “Cellu Sep” cellulose tubular membrane with a molecular weight cut‐off (MWCO) of 3.5 kDa against HEPES lysis buffer at 4°C for 6 hr. A 20‐fold molar excess of MTSSL (MW = 264.3 g/mol) was added to the Cu(I)‐CueR solution in a 1:20 = [Cu(I)‐CueR]:[spin‐label] ratio, and then mixed overnight at 4°C protected while from light. The free spin‐label was removed by dialysis at 4°C for 1 day. This procedure ensures that no free spin‐label was left, and that solely the selected cysteine residues are labeled. Moreover, during the centrifugation steps, all Cu(I) was removed from the protein. CueR protein was concentrated and quantified by a NanoDrop spectrophotometer. The final concentration of CueR protein after spin labeling was 40–60 μM.

### 2.5 Cu(I) addition

After spin‐labelling and all the dialysis steps carried out for removal of free spin‐labels from the solution, no Cu(I) ions were found in the protein solution. This was verified by adding 0.1 mM KCN to the solution and acquiring the CW‐EPR spectra. The spectra in the presence and absence of KCN were similar.

For EPR measurements, Cu(I) (Tetrakis (acetonitrile) copper(I) hexafluorophosphate) was added to the protein solution under nitrogen gas to preserve anaerobic conditions. No Cu(II) EPR signal was observed at any time.

### 2.6 CW EPR measurements

CW‐EPR (Continuous wave EPR) spectra were recorded using E500 Elexsys Bruker spectrometer operating at 9.0–9.5GHz. The spectra were recorded at room temperature at microwave power of 20.0 mW, modulation amplitude of 1.0 G, a time constant of 60 ms, and receiver gain of 60.0 dB. The samples were measured in 0.8 mm capillary quartz tubes (Vitrocom).

### 2.7 CW‐EPR simulations

A CW‐EPR spectrum of CueR_A16R1 at 130 K was simulated using EasySpin^22^ to determine the *g* and A values for the RTsimulations. The values were: *g*_xx_ = 2.0088, *g*_yy_ = 2.0058, *g*_zz_ = 2.0028, *A*_xx_ = 16 MHz, *A*_yy_ = 16 MHz, and *A*_zz_ = 105 MHz. The RT CueR data were simulated using the chili function in EasySpin with these values. Each spectrum was simulated with a two‐component fit. The *β*_D_ parameter for each component was constant at 15°. For each mutant, the exact values of each parameter are provided in Tables S1–S3. For the M101R1 mutant, both order parameter and population weight of the immobile component were varied to fit the spectra. For the A16R1 mutant, only rotational correlation time was varied. For the G57R1 mutant, only the population weights of the components were varied.

### 2.8 DEER measurements

The DEER experiment *π*/2(*ν*_obs_) − *τ*1 – *π* (*ν*_obs_) − *t*′ – *π* (*ν*_pump_) − (*τ*1 + *τ*2 − *t*′) − *π*(*ν*_obs_) − *τ*2 − echo was carried out at 50 ± 1.0 K on a Q‐band Elexsys E580 spectrometer (equipped with a 2‐mm probe head). A two‐step phase cycle was employed on the first pulse. The echo was measured as a function of *t*′, whereas *τ*2 was kept constant to eliminate relaxation effects. The durations of the observer *π*/2 and *π* pulses were 12 and 24 ns, respectively. The duration of the π pump pulse was 24 ns, and the dwell time was 16 ns. *τ*1 was set to 200 ns. The observer frequency was 33.73 GHz, the pump frequency was 33.79 GHz, and the magnetic field was 12,020 G. The samples were measured in 1.6 mm capillary quartz tubes (Wilmad‐LabGlass). The data were analyzed using the DeerAnalysis 2019 program^23^ and DEERNet.^24^

### 2.9 Circular dichroism characterization

Circular dichroism (CD) measurements were performed at RT using a Chirascan spectrometer (Applied Photophysics, UK). Measurements were carried out in a 1 mm optical path length cell. The data were recorded from 190 to 260 nm with a step size and a bandwidth of 1 nm. Spectra were obtained after background subtraction.

## 3 RESULTS

Room temperature (RT) CW‐EPR experiments coupled with nitroxide spin‐labeling have been used for many years to study biological systems.^25^, ^26^, ^27^, ^28^ The basic idea is to site‐specifically label a biological macromolecule with a small free radical and then measure the mobility of the spin‐label from the EPR lineshape. For surface‐exposed helical sites and loops, changes in EPR lineshapes can be attributed to changes in site‐specific backbone dynamics, which then can be related to biological function.^27^, ^28^, ^29^ The most common spin‐label is S‐(2,2,5,5‐tetramethyl‐2,5‐dihydro‐1H‐pyrrol‐3‐methyl) methanethiosulfonothiate (MTSSL) which is chemically attached to cysteine residues in the protein and causes minimal perturbation to the protein.^30^, ^31^

**FIGURE 2:**
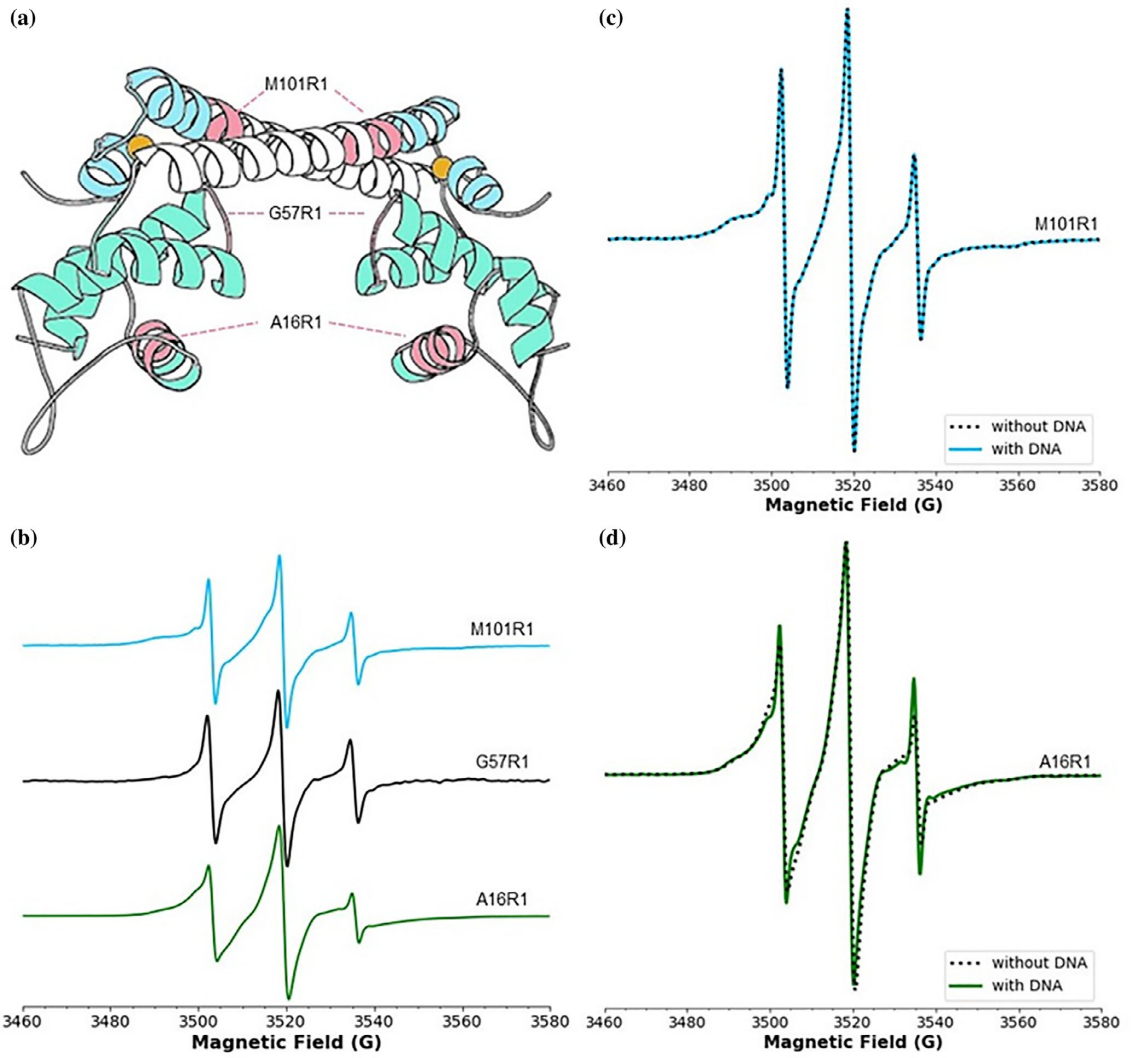
(A) Nitroxide spin‐labelled sites in *Escherichia coli* CueR. The different sites are marked by pink on the CueR (PDB 1q05) structure. (B) RT CW‐EPR spectra for three different mutants: CueR_M101R1, CueR_G57R1, and CueR_A16R1, in the apo‐state. (C) RT CW‐EPR spectra for CueR_M101R1 mutant in the absence and presence of DNA. (D) RT CW‐EPR spectra for CueR_A16R1 mutant in the absence and presence of DNA

In this work, we exploited this site‐directed spin‐labeling methods to obtain site‐specific information on protein dynamics. The *E. coli* CueR has four cysteine residues: C112 and C120 which compose the Cu(I) binding site, and C129 and C130 at the C‐terminal domain. To avoid spin‐labeling at the Cu(I) site, we developed a methodology of spin‐labeling in the presence of Cu(I) as described in our previous studies^17^, ^18^ and in the methods section. The bound Cu(I) ion was removed by a series of dialysis steps, and we verified that no Cu(I) was remaining using experiments with KCN. We preserved C112 and C120 but we mutated C129 and C130 to alanine residues. We then expressed three different mutants (Figure 2a): CueR_M101C (α5 dimerization helix, close to the Cu(I) binding site), CueR_G57C (on a loop between α3 and α4), and CueR_A16C (α2 helix, in the DNA binding domain). After spin‐labeling with MTSSL, we termed these spin‐labeled sites as R1, as is customary in literature. EMSA and CD (see Figures S1 and S2) confirmed that the spin‐labeling and point mutations did not affect the protein–DNA interaction and the secondary structure of the protein. All EPR experiments preformed here were done under anaerobic conditions.

Figure 2B shows the CW‐EPR spectra for the three different mutants in the apo‐state. A visual examination of the apo‐state shows profound differences in site‐specific dynamics at different sites. These differences are most prominent in the low‐field part of the spectrum (below ca. 3,500 G). For example, the M101R1 has a broad component that persists to ca. 3,490 G, which is indicative of slow backbone reorientation. On the other hand, G57R1 has the narrowest linewidth, indicative of faster motion. Since G57R1 is on the loop between two helices, faster dynamics for this site are expected. Finally, A16R1 has broader lines than G57R1, which is indicative of slower dynamics. The dynamics of A16R1 site are restricted, owing to its location in the packed DNA binding domain comprising of four helices and two beta sheets. Qualitatively, these data illustrate the utility of R1 labeling to measure site‐specific dynamics in this protein. Figure 2C,D also shows CW‐EPR data for M101R1 and A16R1 in the presence and absence of DNA. The CW‐spectra of free protein and the DNA‐bound protein are virtually identical, which further validate that the effects of global tumbling are negligible and that the lineshapes are dominated by site‐specific dynamics. More importantly, these data suggest that any changes in lineshape upon addition of Cu(I), can be attributed to changes in the site‐specific dynamics due to Cu(I) binding.

**FIGURE 3:**
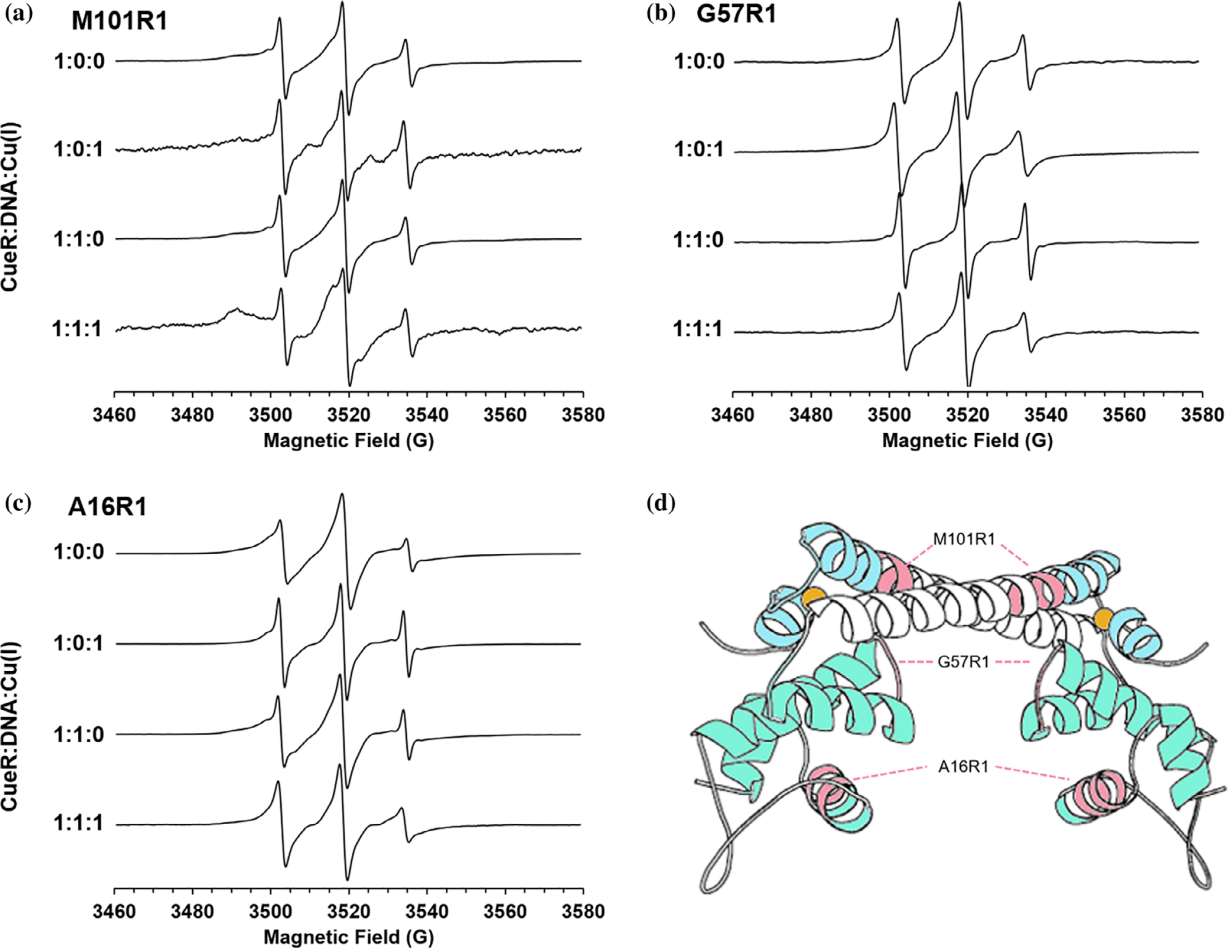
RT CW‐EPR spectra for three different mutants: (A) CueR_M101R1, (B) CueR_G57R1, and (C) CueR_A16R1, at four different states: apo‐CueR, holo‐CueR (in the presence of Cu(I), 1:0:1 ratio), repressed CueR (in the presence of DNA, 1:1:0 ratio), and active CueR (in presence of Cu(I) and DNA, 1:1:1 ratio)

Next, we collected the data for each mutant in three different functional states. The spectra of the different mutants in presence of an equivalent amount of Cu(I) per monomer (holo‐state), CueR:DNA complex (repressed state, 1:1 ratio), and CueR:DNA:Cu(I) complex (active state, 1:1:1 ratio) are shown in Figure 3 (the ratio described in Figure 3 is per CueR monomer so a 1:1 CueR:Cu(I) ratio implies one Cu(I) per monomer). Note, there are changes in lineshapes for apo‐CueR versus holo‐CueR, and repressed versus active‐states of the protein. Expectedly, these changes are most dramatic for M101R1 and A16R1, where the labels are positioned on helical sites. More importantly, these data indicate that the binding of Cu(I) induces large changes in site‐specific protein dynamics in both the DNA‐free (apo vs holo) and the DNA‐bound (repressed vs active) states.

In order to further explore these dynamical effects, we performed systematic experiments where we measured CW‐EPR spectra as a function of Cu(I) concentration in both the protein‐only and in the protein–DNA complexes for CueR_M101R1 and CueR_A16R1 mutants. Given the *K*_D_ of 10^−21^ M of Cu(I) to CueR,^6^ we anticipate stoichiometric loading of Cu(I) onto the protein.

**FIGURE 4:**
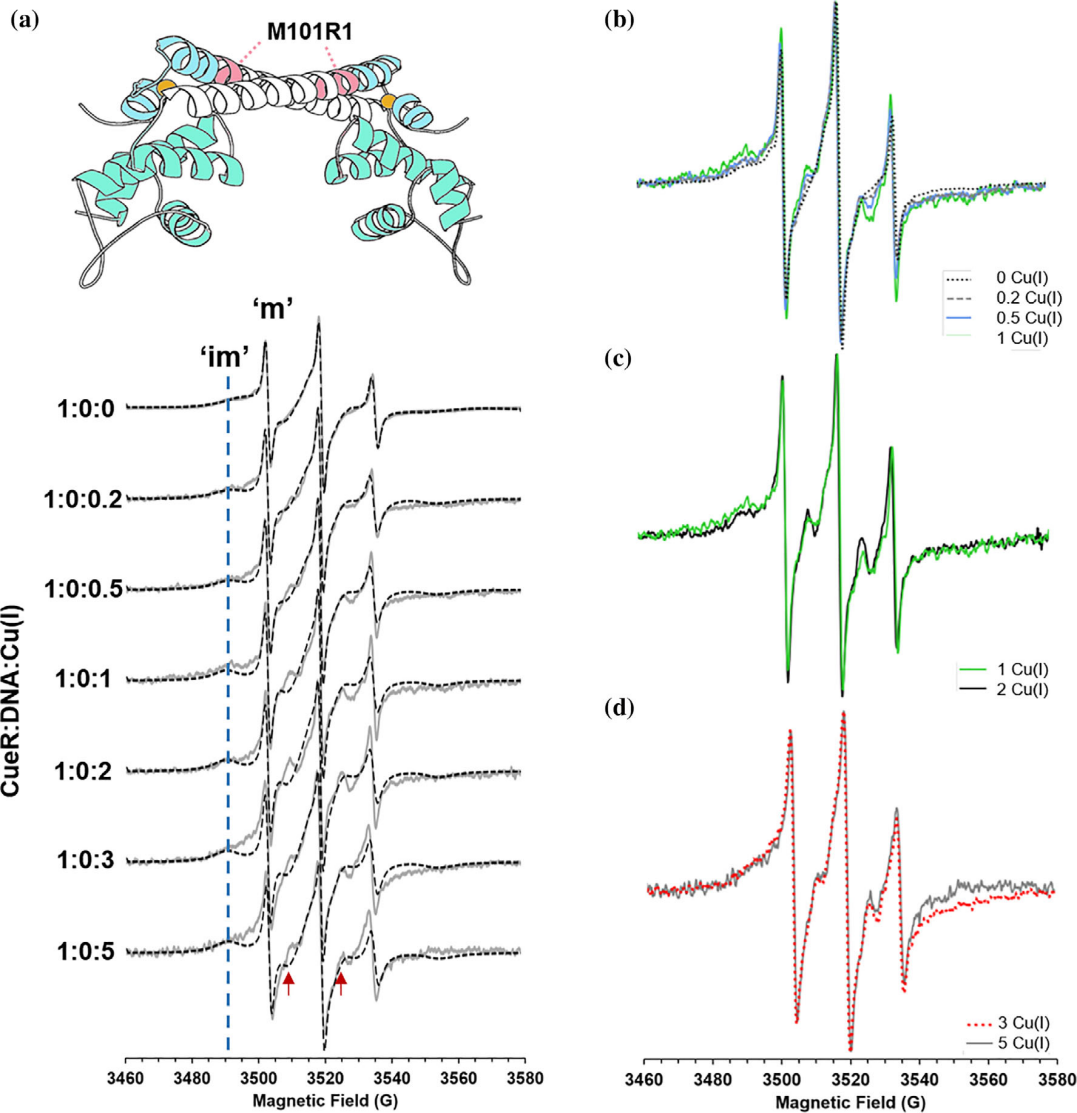
(A) RT CW‐EPR spectra (solid gray lines) for CueR_M101R1 mutant in absence of DNA as a function of different Cu(I) concentrations. Dashed black lines represent the fitted spectra. Fitting parameters are listed in Table S1. The blue dashed line marks the broadening corresponding to the immobile component at 3490 G. The red arrows mark signals that originated from an increase in the order parameter for the immobile component. (B) RT CW‐EPR spectra for four different Cu(I) concentrations in the range of 0–1 vs. CueR monomer concentration. (C) RT CW‐EPR spectra for 1:1 and 2:1 Cu(I):CueR ratio. (D) RT CW‐EPR spectra for 3:1 and 5:1 Cu(I):CueR ratio

The spectrum of the M101R1 mutant (Figure 4a) exhibits a two‐component spectrum, characterized by a mobile (marked as “m” in Figure 4A) and an immobile component (marked as “im” in Figure 4A)—such complex lineshape is not unusual for R1 attached to proteins.^32^, ^33^, ^34^ Figures 4B–D show overlays of the experimental data at various Cu(I) concentrations. Between 0 to 1 equivalents of Cu(I), we observe systematic changes in the lineshapes (Figure 4B), whereas the lineshape is stable between 1 and 2 Cu(I):CueR (Figure 4C), and in presence of excess Cu(I), at a ratio above 3 Cu(I):CueR (Figure 4D).

The lineshape was analyzed using the microscopic order macroscopic disorder (MOMD) model^28^, ^35^ as implemented in EasySpin.^22^ The simulation parameters are given in Tables S1 and S2. Earlier work^36^ has shown that at surface exposed helical sites, the dynamics of the R1 side chain are coupled with backbone motions, and the rotational correlation times and ordering parameters are conserved for rigid sites.^37^ An increase in site‐specific dynamics results in a decrease in the order parameter and/or in the characteristic correlation time. The analysis of the slow motional lineshape is complex since the parameters are correlated and, in this case, there are additional adjustable parameters due to the presence of two components. For this reason, we followed a conservative approach. We obtained the g‐ and hyperfine parameters from a low‐temperature measurement (Figure S3). The values are consistent with those observed for R1 in proteins.^28^ We used the 1:0:0 dataset to obtain the basis correlation times, weights, and coefficient for the order parameters for each mutant. For the M101R1 mutant, we found that an order parameter was essential to capture the spectral features. For subsequent spectra as a function of added Cu(I) (and with or without DNA) for M101R1 mutant, we only varied the coefficient for the order parameter of the dominant species and the weight for this component. A reasonable, but not perfect, simulation was achievable for all datasets for the M101R1 mutant using this approach.

**FIGURE 5:**
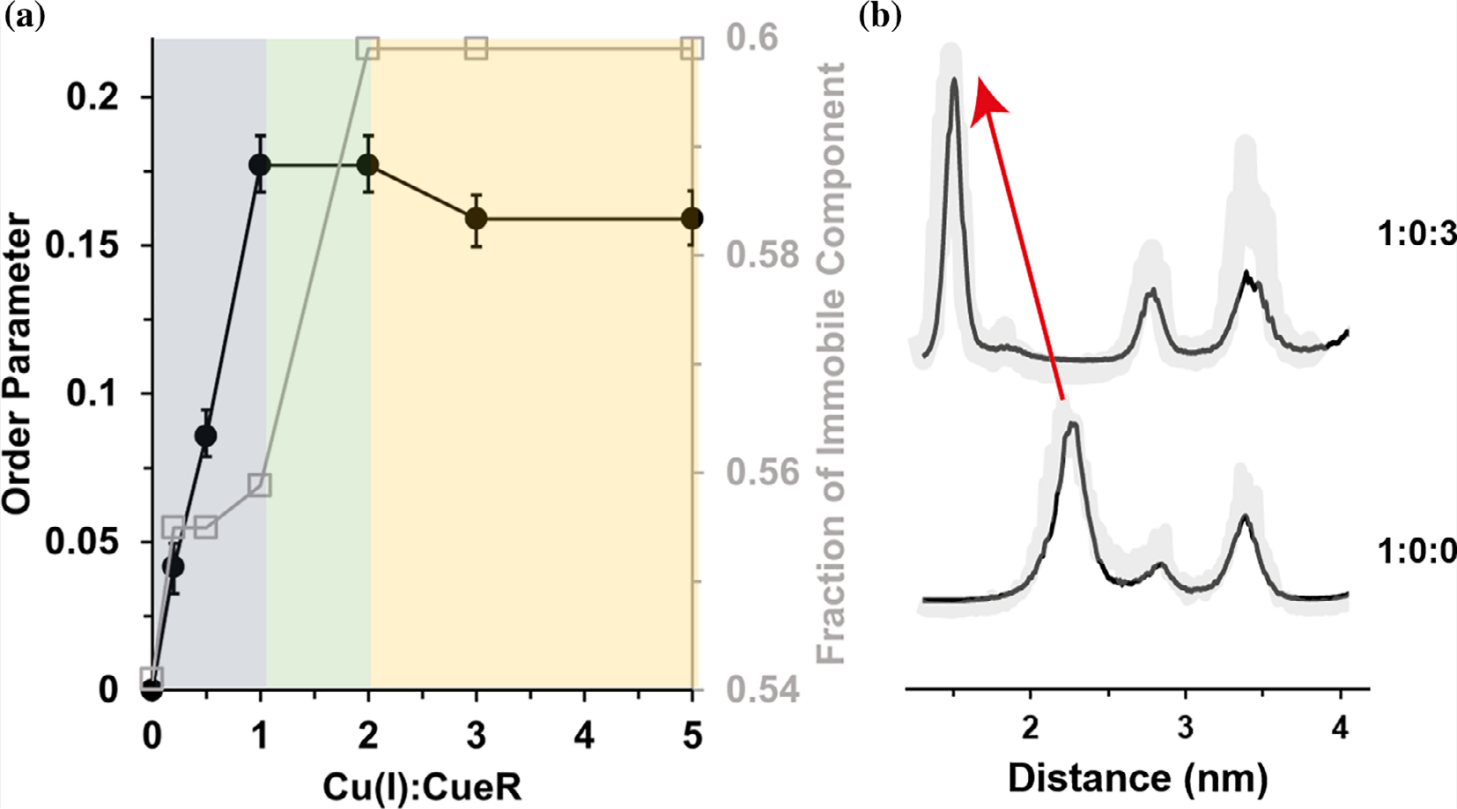
(A) Change in the order parameter of the immobile component (black squares), and the weight of immobile component (gray squares) of CueR_M101R1. (B) DEER distance distribution functions obtained using DEERNet analysis for apo‐CueR and holo‐CueR (1:3 CueR:Cu(I) ratio). The error validation is marked by light gray. The red arrow marks the change in the dominate distance distribution. The time domain DEER signals are presented in Figure S4

Figure 5A presents the changes in order parameter and weight of the immobile component as a function of number of Cu(I) ions per monomer. As the number of Cu(I) ions is increased from 0 to 1, an increase in the order parameter from 0 to 0.18 is detected, which suggests a slowing of site‐specific dynamics. At a higher ratio of Cu(I), between 1–2 Cu(I):CueR, no change in the order parameter was detected, however, there is an increase in contribution of the immobile component from 0.56 at 1:1 Cu(I):CueR to 0.6 at 2:1 Cu(I):CueR ratio. In presence of excess Cu(I), there is a slight decrease in order parameter to 0.16; however, no change in the contribution of the immobile component was detected. This result indicates that binding of the monovalent ion leads to a slowing of backbone motion at this site, which localizes on the Cu(I) binding domain of the protein. We also performed double electron–electron resonance (DEER) experiments (the time domain signals are presented in Figures S4) to evaluate the distances between the spin‐labels by measuring the interspin dipolar interaction.^38^ Indeed, these experiments showed a decrease in the average distance from 2.3 nm in the apo‐state to 1.6 nm in the holo‐state. More importantly, there is a decrease in the width of the distribution from 0.3 nm to 0.1 nm, which agrees well with the increase in the order parameter and decrease in mobility of the M101R1 site (Figure 5b).

**FIGURE 6:**
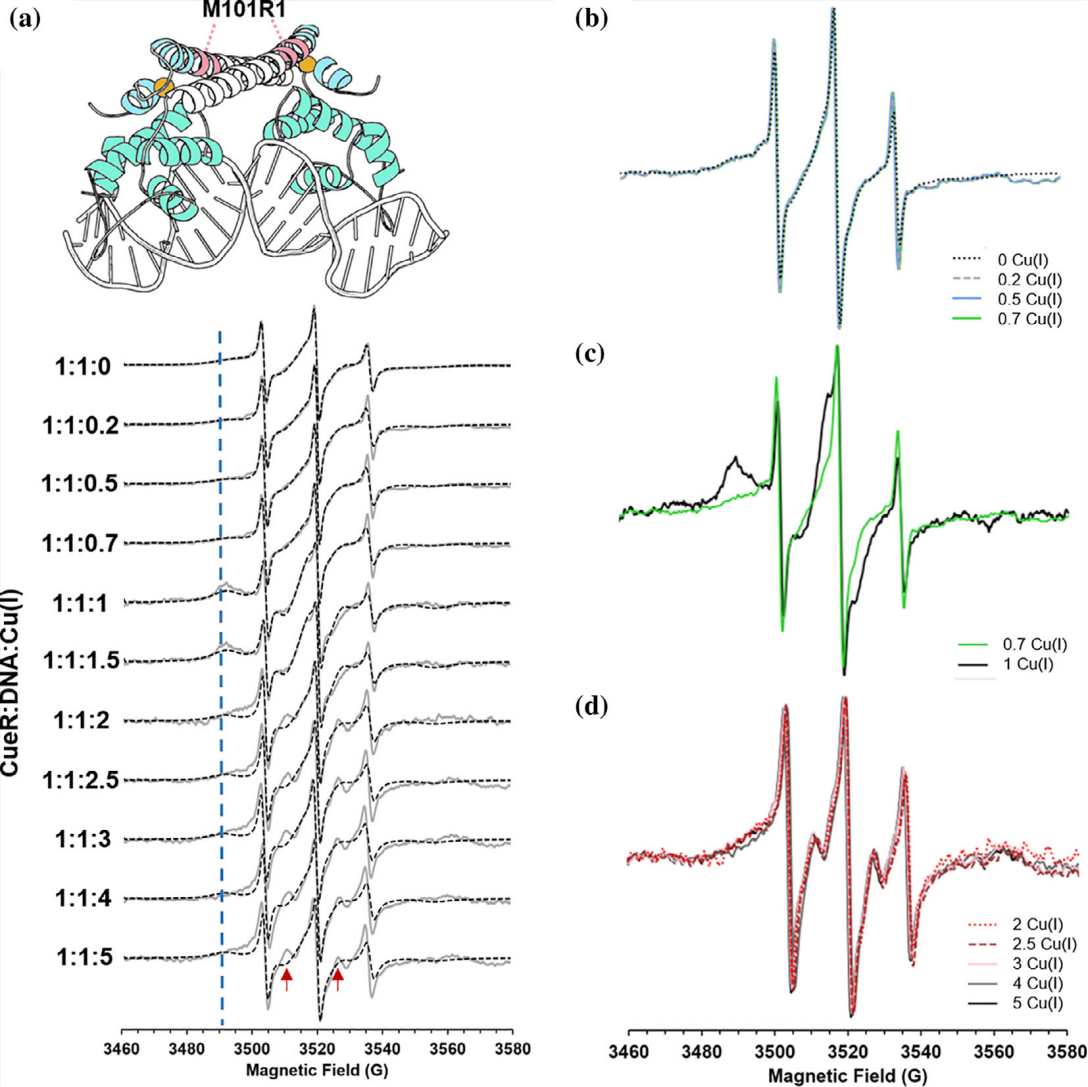
(A) RT CW‐EPR spectra (solid gray lines) for CueR_M101R1 mutant in presence of DNA as a function of different Cu(I) concentrations. Dashed black lines represent the fitted spectra. Fitting parameters are listed in Table S1. The blue dashed line $\tau_{\text{corr}}^{-1}$ marks the broadening that corresponds to the immobile component at 3490 G. The red arrows mark signals originating from an increase in the order parameter for the immobile component. (B) RT CW‐EPR spectra for four different Cu(I) concentrations in the range of 0–0.7 vs. CueR monomer concentration. (C) RT CW‐EPR spectra for 1:1:0.7 and 1:1:1 Cu(I):CueR:DNA ratio. (D) RT CW‐EPR spectra for Cu(I) concentrations in the range of 2–5 vs. CueR monomer concentration

Next, we examined the effect on dynamics of M101R1 in the presence of DNA. The data are shown in Figure 6a. Note the 0‐equivalent of Cu(I) (top‐most spectrum) corresponds to the repressed state and the system transitions to the active state as the number of Cu(I) ions increases. Qualitatively, the effect of Cu(I) on dynamics in the presence of DNA at this site is larger than in the absence of DNA. These results are intriguing given that the site is far from the DNA binding domain. The experimental data suggest three different states. The first state is at Cu(I):CueR:DNA of 0–0.7:1:1 (Figure 6b) where the lineshape is stable. The second state between 0.7–1 Cu(I):CueR:DNA (Figure 6C) exhibits dramatic changes in the spectral shape, and in the third state, in presence of excess Cu(I), at a ratio above 2 Cu(I):CueR:DNA (Figure 6D), the lineshape becomes stable again.

**FIGURE 7:**
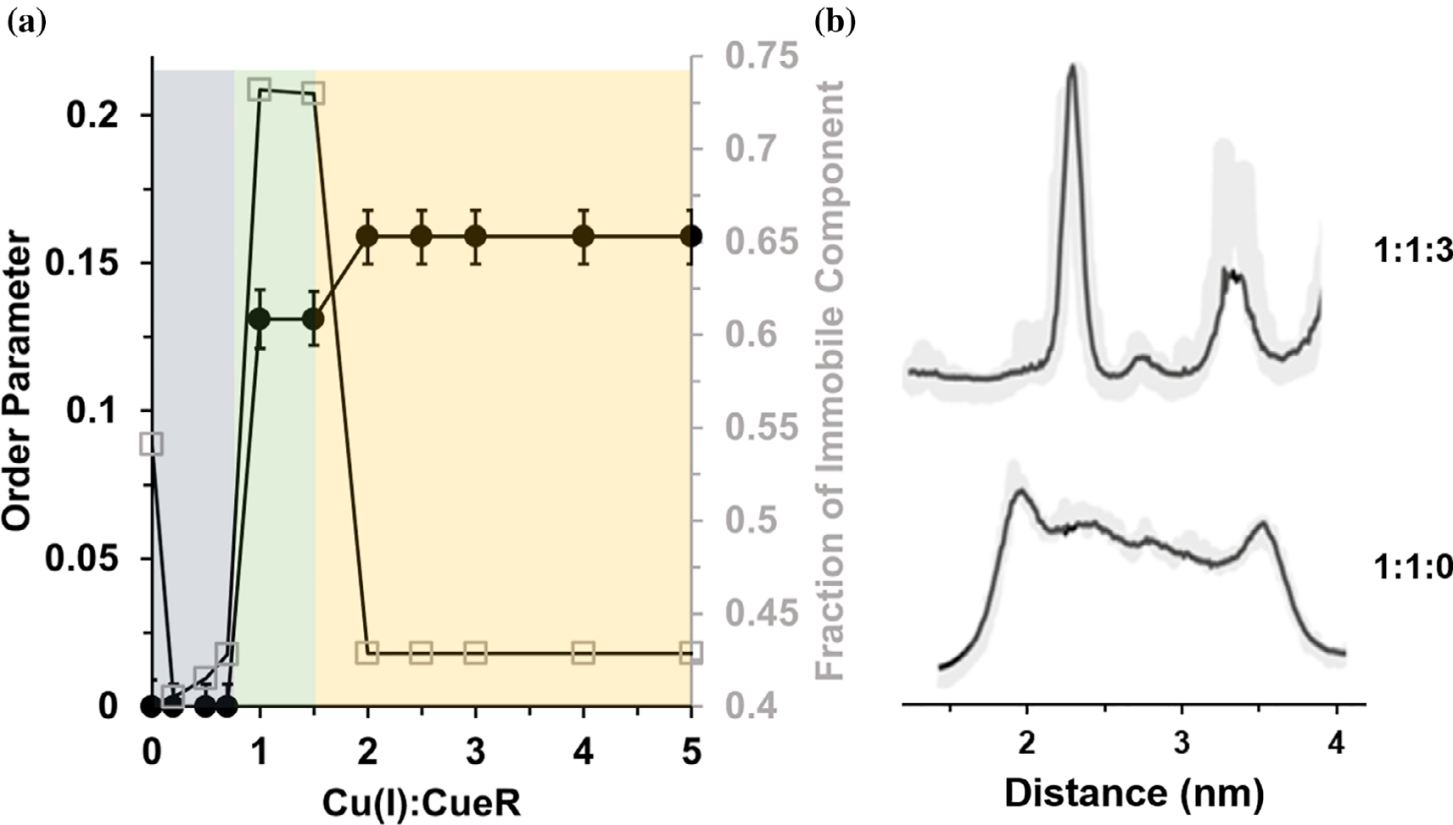
(A) Change in the order parameter of the immobile component, (black squares), and the weight of the immobile component (gray squares) of CueR_M101R1 in the presence of DNA. (B) DEER distance distribution functions obtained using DEERNet analysis. The error validation is marked by light gray. The time domain DEER signals are presented in Figure S4

Figure 7a presents the changes in order parameter and weight of the immobile component as a function of the number of Cu(I) ions per monomer. In the presence of Cu(I), there is no noticeable change in dynamics until 0.7 equivalent of Cu(I) is added. There is a sharp increase in the order parameter between 0.7 and 1 equivalent of Cu(I). In addition, there is a large increase in the fraction of this component (from 0.41 to 0.73). After about 2 equivalents of Cu(I) are added, the order parameter changes only slightly but the fraction of the immobile component decreases. Together, these results suggest that when one Cu(I) is bound to each protein site in the presence of DNA, the dynamics at this site become restricted. However, an increase in Cu(I) concentration leads to a partial release of this tight CueR‐DNA structure. Changes in the conformation of the α5 helix, also led to a change in the distance distribution between the spin‐labels in the repressed and active states (Figure 7b). While in the repressed state, a broad distance distribution was obtained due to higher flexibility and multiple conformations, while in the active state, a narrow distance distribution was observed.

**FIGURE 8:**
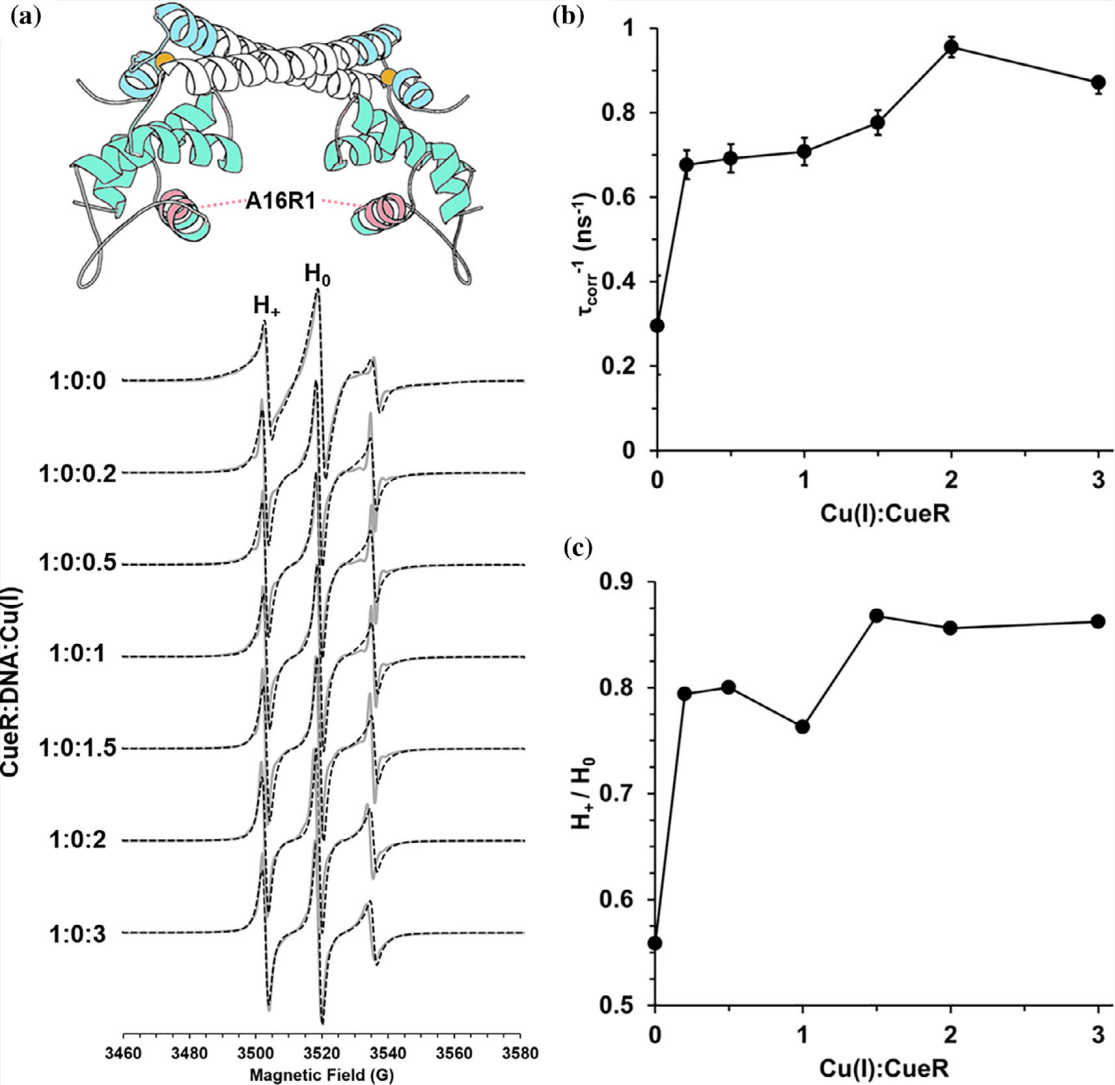
(A) RT CW‐EPR spectra (solid gray lines) for CueR_A16R1 mutant in the absence of DNA as a function of different Cu(I) concentrations. Dashed lines represent fitted spectra. Fitting parameters are listed in Table S2. (B) The change in $\tau_{\text{corr}}^{-1}$, inverse rotational correlation time as a function of [Cu(I)]. C. The change in the intensities H_+_/H_0_ as a function of [Cu(I)]

**FIGURE 9:**
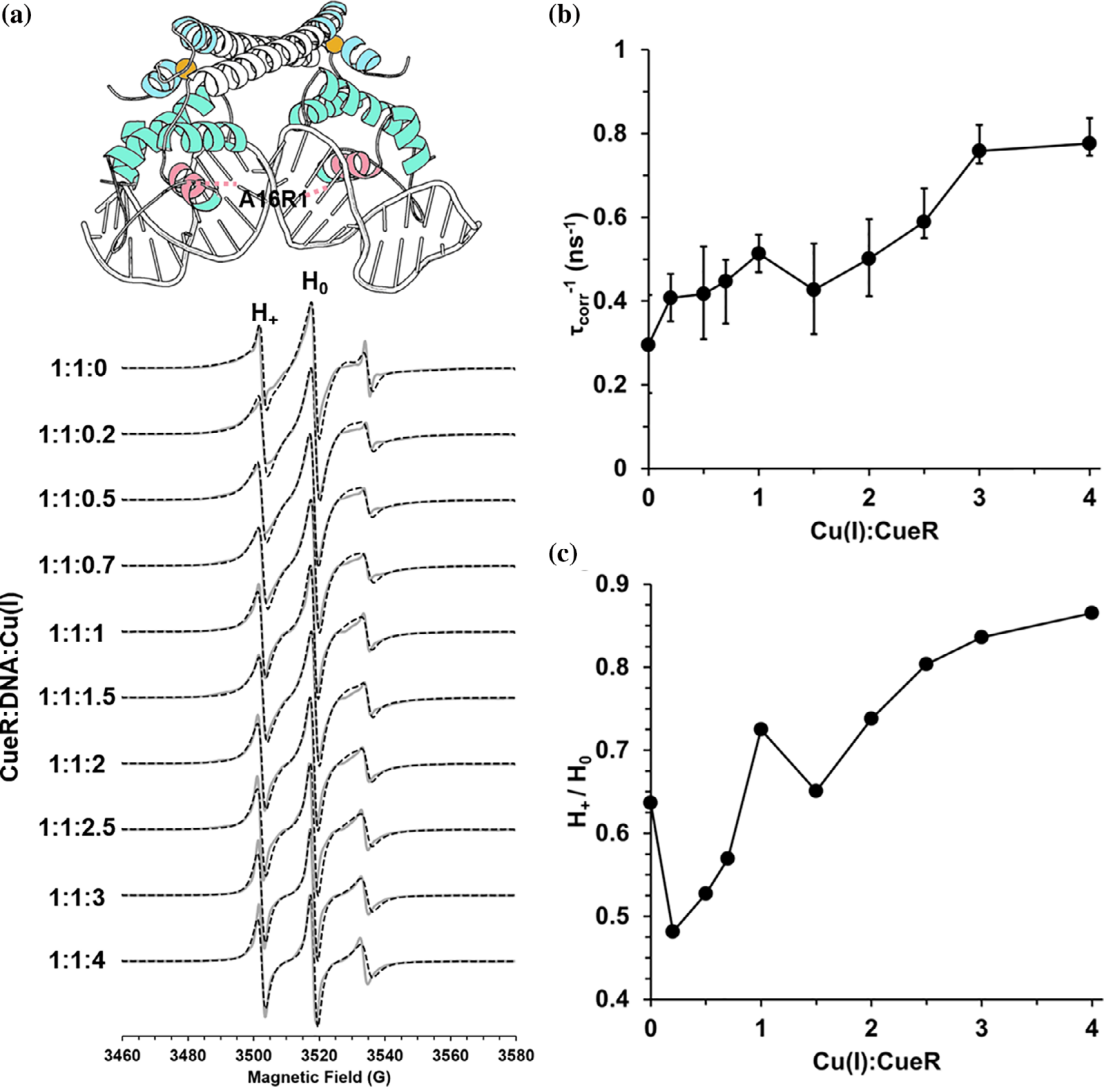
(A) RT CW‐EPR spectra (solid gray lines) for CueR_A16R1 mutant in the presence of DNA as a function of different Cu(I) concentrations. Dashed lines represent fitted spectra. Fitting parameters are listed in Table S2. (B) The change in $\tau_{\text{corr}}^{-1}$, inverse rotational correlation time as a function of [Cu(I)]. (C) The change in the intensities H_+_/H_0_ as a function of [Cu(I)]

We performed similar experiments for the CueR_A16R1 mutant (Figures 8 and 9), which is located at the DNA binding domain. For these simulations, an order parameter was not needed for either component. Therefore, we only varied the correlation time of the dominant species and kept the weights constant. Figure 8a shows the CW‐EPR spectra (solid gray lines) and the fitted spectra (dashed black lines) for CueR_A16R1 in the absence of DNA. Figure 8b shows the change in correlation time as a function of [Cu(I)] for CueR_A16R1 mutant in the absence of DNA. Upon addition of 0.2Cu(I):CueR, there is an immediate increase in the dynamics of the spin‐label. At higher [Cu(I)], further increase in the dynamics is detected. We also measured the ratio between the intensity of H_+_(lower field absorption, corresponds to *m*_I_ = +1)/ H_0_(central field absorption, corresponds to *m*_I_ = 0). An increase in this ratio corresponds to higher dynamics. Figure 8C shows similar trends as Figure 8B, supporting an increase in dynamics at the A16R1 site upon Cu(I) binding. In the presence of DNA (Figure 9), a gradual increase in dynamics is detected as a function of [Cu(I)]. CW‐EPR experiments and simulations for CueR_G57R1 mutant are presented in Figure S5. For this mutant, only variation in the immobile component was required as a function of Cu(I) binding.

In summary, the EPR experiments suggest that upon Cu(I) binding, the dynamics of CueR near the Cu(I) binding site decrease, while there is an increase in dynamics at the DNA binding domain. In presence of DNA, the lowest dynamics of the overall protein were detected at a ratio of 1:1:1 Cu(I):CueR:DNA. At higher Cu(I) concentration, the dynamics of CueR increase at both the Cu(I) binding site and the DNA binding site.

## 4 DISCUSSION

In this study, we investigated the role of Cu(I) and DNA in the allosteric mechanism of the *E. coli* CueR transcription regulation. CueR is found in many Gram‐negative bacteria aside from *E. coli*; CueR homologs are found in pathogenic systems such as *Salmonella typhimurium* or *Pseudomonas aeruginosa*. In all bacteria, CueR shares a sequence similarity above 50%. As metal ions are essential for many bacterial reactions and are vital for nearly all aspects of bacterial metabolism, metal homeostasis is a promising target for antibacterial drug development. Therefore, understanding the mechanism of action of bacterial metalloregulators can serve as a basis for the development of novel mechanism‐based antibiotics linked to deregulation of copper metabolism.

The function of CueR depends intimately on allosteric changes in structure and dynamics that result from Cu(I) and DNA binding. Allostery is a fundamental thermodynamic phenomenon in which the binding of a specific ligand influences the activity of a biomolecule. A thorough understanding of allostery is challenging due to difficulties in monitoring numerous and different conformational states during the biological process in solution.^39^ Previous studies provided a comprehensive understanding of the transcription initiation mechanism involving the MerR family monomer (EcmrR), DNA and entire RNAP complex at different stages of the initiation process measured employing cryo EM.^40^ Here, we applied RT CW‐EPR measurements and site‐directed spin labeling to follow dynamic changes in CueR upon Cu(I) and DNA binding. We focused on two domains of the CueR protein: α5 helix close to the Cu(I) binding site, by spin‐labeling M101 residue, and the DNA binding domain, α2 helix, by spin‐labeling A16 residue.

Close to the Cu(I) binding site (M101R1 site), we observed a progressive decrease in dynamics upon the addition of Cu(I), in the absence of DNA. This is expected since the high‐affinity binding site of Cu(I) involves two adjacent helices α5 and α6, that are connected via linear Cu(I) dithiolate coordination between C112 (α5) and C120 (α6) in presence of Cu(I) (Figure 1). On the other hand, there are differences in the dynamics in the presence of DNA, even though the M101 site is distant from the DNA binding domain. In the presence of DNA, we observed three different states for the M101R1 site. The first state is described by less than one Cu(I) ion per CueR monomer. In this state, the site is characterized by high dynamics. The second state is prominent while one‐to‐two Cu(I) ions are bound to one CueR monomer. In this state, the dynamics of the protein are tremendously reduced at the Cu(I) binding site. The third state is characterized by larger dynamics compared to the second state, and in this state, two Cu(I) ions can coordinate to one CueR monomer.

**FIGURE 10:**
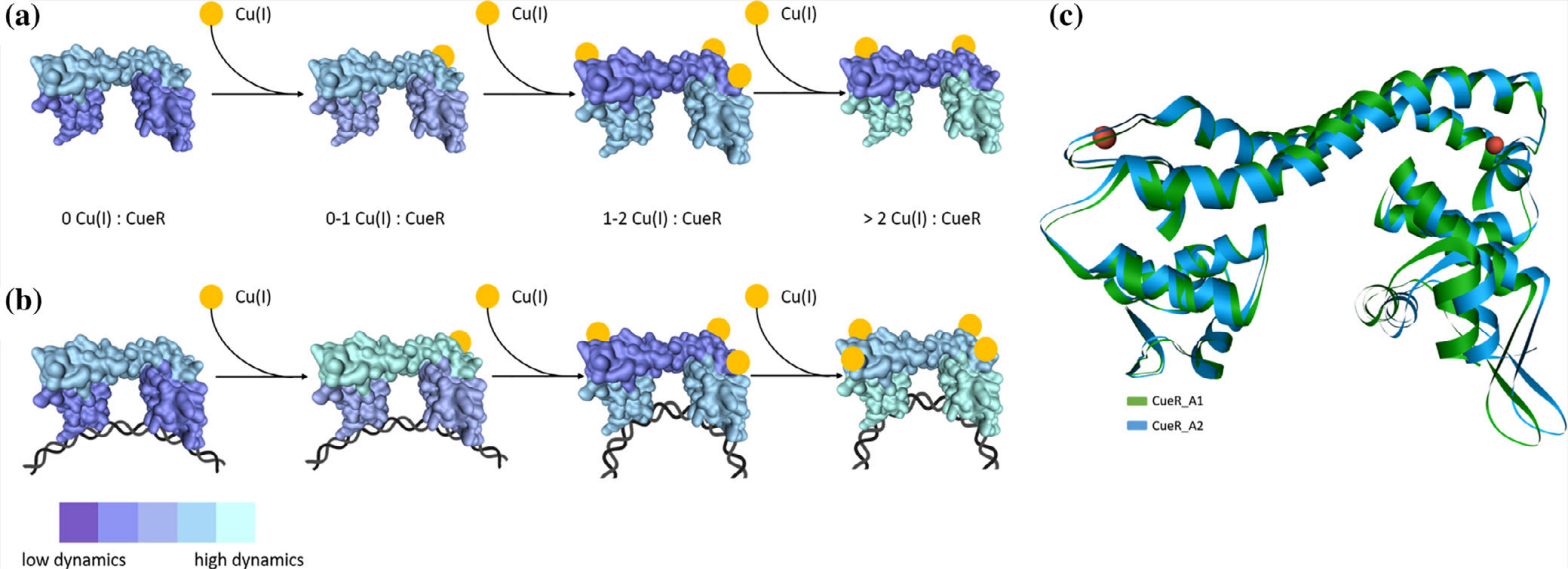
Qualitative model illustrating the dynamical changes CueR undergoes upon Cu(i) binding in absence of DNA (A) and in presence of DNA (B). The orange circles represent the Cu(I) ion. Changes in dynamics are colored from dark to bright while dark colors indicate less dynamics compared to lighter colors. The color coding represents the dynamics within one domain and state (DNA bound) and not between domains or states. (C) The two active state conformations resolved using EPR constraints as reported in ref.^17^

More interesting are the changes in dynamics at the A16R1 site, which is more than 27 Å away from the high‐affinity Cu(I) binding site. The progressive binding of Cu(I) leads to an increase in dynamics in the DNA binding domain in the presence and absence of DNA. The fluctuations of the DNA binding region play a critical role in recognition of the specific DNA sequence, and correlation of the protein fluctuation with the DNA fluctuations is critical for the formation of protein–DNA contacts that lead to binding and bending of the DNA. This data show, for the first time, that Cu(I) binding triggers changes in dynamics distant from the Cu(I) binding site, at the DNA binding domain. This data also provide more insight into the previous EPR results that probed the DNA conformation in different functional states.^11^ There we showed that apo‐CueR can bend the DNA but a large excess of CueR is required. In the presence of Cu(I), DNA bending occurs at lower CueR concentration. Mechanistically, a rapid modulation of dynamics of the DNA binding domain is critical even for apo‐CueR since fast binding to DNA is critical in order to initiate transcription and relieve the cell from copper stress. Figure 10 summarizes the change in the dynamics f CueR as a function of Cu(I) binding.

Previously, we preformed DEER measurements at various sites of CueR (G11R1 between helices α1 and α2) and L60H_G64H on helix α4.^17^, ^18^, ^41^ These experiments suggested two different conformational states of CueR for the active state, in presence of Cu(I) and DNA. Differences between these two states were detected upon varying Cu(I) concentrations. We named the conformation of the active state when one‐to‐two Cu(I) were bound to CueR monomer as A1, and the conformation at higher Cu(I) concentration as A2 (see Figure 10C). A1 is characterized by a smaller distance between the two DNA binding domains (Glu33_Cα^A^‐ Glu33_Cα^B^ = 6.2 nm), while A2 is characterized by a slightly longer distance between the two DNA binding domains (Glu33_Cα^A^‐ Glu33_Cα^B^ = 6.32 nm). The data published earlier about the “dead‐end” conformation,^16^ together with the EPR results reported in this study on the dynamical changes in M101R1 site (Figure 6), suggest that A1 is less dynamic and more compact. It is likely that the A1 conformation can bend the DNA promoter sequence to a larger extent than the A2 conformation, which might indicate that this is indeed the conformation that initiates the transcription process. At higher Cu(I) concentrations, inhibition of this process occurs, previously reported as a “dead‐end” state by smFRET experiments.^16^ Therefore, at higher copper concentrations, alternative copper regulation mechanisms controlled by other copper regulators could reduce prolonged and elevated copper levels.^2^

In our previous studies,^17^, ^18^ we also hypothesized that there is more than one Cu(I) binding site in CueR. Recently, Balogh et al.^42^ explored the binding of Hg(II) to CueR and suggested that the last seven residues at the C‐terminal tail of CueR form a second metal ion binding site with C129 and C130 coordination. Their conclusions were made based on mass spectroscopy analysis of WT‐CueR and truncated Δ7‐CueR (where the last seven residues CCHHRAG were missing). In this study, the C‐terminal tail was mutated to AAHHRAG. Therefore, C129 and C130 cannot form a second binding site, but the C‐terminal histidine residues might form a second Cu(I) ion binding site, albeit not conserved throughout the CueR protein family. Similar findings were reported for the CAP monomer which is also capable of binding two cAMP molecules per monomer further supporting our conclusion of multiple binding sites for Cu(I) in CueR.^19^ Further studies are required to fully understand the role of the C‐terminal histidine and cysteine residues of Cu(I) binding in CueR.

## 5 CONCLUSIONS

In this work, we show that room‐temperature CW‐EPR experiments together with site‐directed spin‐labeling can resolve the dynamical changes in CueR upon Cu(I) and DNA binding. These experiments shed light on the role of Cu(I) in the transcription mechanism of CueR. Different states of the protein were detected upon Cu(I) binding which trigger dynamical changes both at the Cu(I) and DNA binding sites. The first state is described by less than one Cu(I) bound to the CueR monomer. The second state is defined upon binding of one‐to‐two Cu(I) ions to the CueR monomer, and the last state is described by two Cu(I) ions bound to each CueR monomer. Higher Cu(I) concentration does not affect the conformation and dynamics of CueR. This indicates that at least two Cu(I) ions can bind to one CueR monomer, and these two sites might also regulate the transcription mechanism. Interestingly, we show that Cu(I) binding leads to an increase in dynamics ca. 27 Å away at the DNA binding domain. These changes in dynamics of the DNA binding domain are important for exact coordination with the DNA. Altogether, the described experimental data in this study provide strong evidence that allosteric changes in dynamics are induced by binding of DNA and Cu(I) and thus advance our understanding of the role of copper in the regulation of transcription by metalloregulator proteins.

## AUTHOR CONTRIBUTIONS

**Idan Yakobov:** Data curation (equal); formal analysis (equal); investigation (equal); writing – original draft (equal). **Alysia Mandato:** Formal analysis (equal); investigation (equal); methodology (equal); writing – original draft (equal); writing – review and editing (equal). **Lukas Hofmman:** Conceptualization (equal); project administration (equal); writing – original draft (equal); writing – review and editing (equal). **Kevin Singewald:** Formal analysis (equal). **Yulia Shenberger:** Investigation (equal). **Lada Gevorkyan‐Airapetov:** Methodology (equal); project administration (equal). **Sharon Ruthstein:** Conceptualization (equal); investigation (equal); methodology (equal); project administration (equal); supervision (equal); writing – review and editing (equal).

## CONFLICT OF INTEREST

The authors declare no conflict of interests.

## Supporting information


**Appendix S1.** Supporting Information
