# Supplementary material for: Allostery‐driven changes in dynamics regulate the activation of bacterial copper transcription factor
Source: Protein Sci. 2022 Apr 12;31(5):e4309. doi: 10.1002/pro.4309 (PMC9004249; doi:10.1002/pro.4309)
Supplement: Supplementary file 1 — Appendix S1. Supporting Information [file PRO-31-e4309-s001.pdf]

Supporting Information

Allostery-driven changes in dynamics regulate the activation of bacterial copper transcription factor

Idan Yakobov[+,1], Alysia Mandato[+,2], Lukas Hofmann[+,1], Kevin Singewald[2], Yulia Shenberger[1], Lada Gevorkyan-Airapetov[1], Sunil Saxena[*,2], Sharon Ruthstein[*,1].

[1] Department of Chemistry and the Institute of Nanotechnology & Advanced Materials, Faculty of exact sciences, Bar Ilan University, Ramat-Gan, Israel, 5290002.

[2] Department of Chemistry, University of Pittsburgh, PA, USA, 15260.

[+] Equal Contribution

[*] Corresponding authors: Sharon.ruthstein@biu.ac.il; sksaxena@pitt.edu.

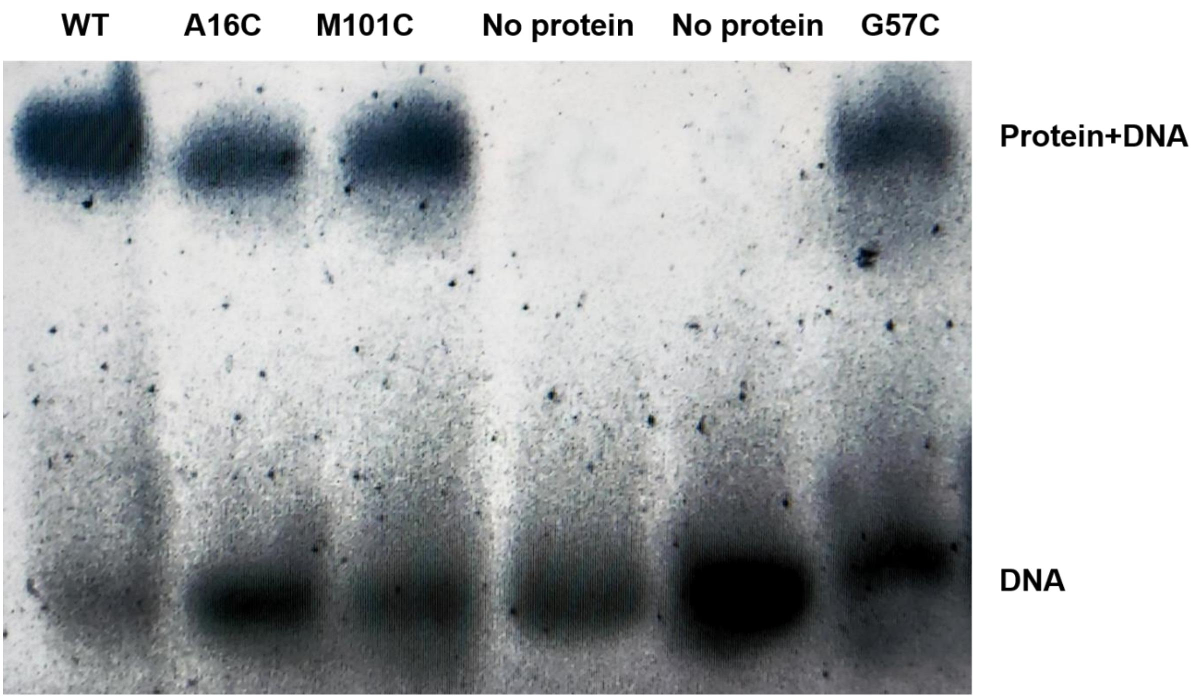

Figure S1: EMSA gel of various CueR mutants.

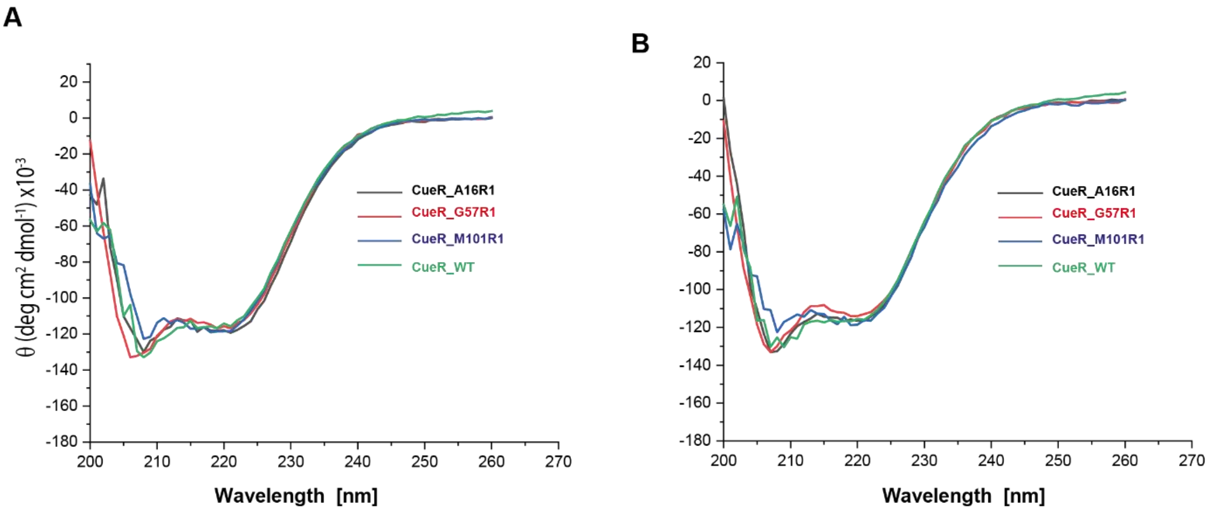

Figure S2: CD spectra of the various spin-labeled CueR mutants and WT-CueR in the absence (**A**) and presence (**B**) of Cu(I).

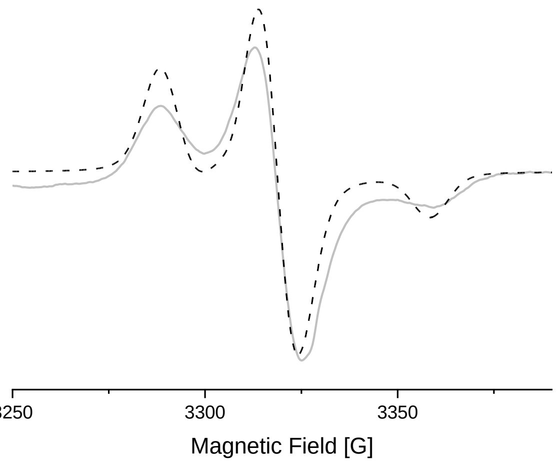

Figure S3: CW-EPR spectrum of CueR_A16R1 at 130K (solid grey line). Dashed black line represents the fitted spectrum with g_{xx} = 2.0088, g_{yy} = 2.0058, and g_{zz} = 2.0028; A_{xx} = 16, A_{yy} = 16, and A_{zz} = 105 MHz.

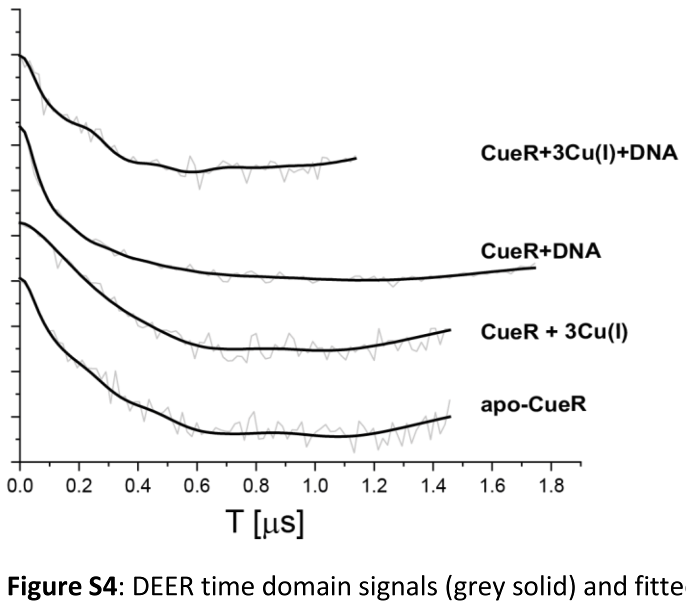

Figure S4: DEER time domain signals (grey solid) and fitted signals (dark solid lines) for CueR_M101R1 mutant.

Table S1: Parameters derived from MOMD simulations for CueR_M101R1

	**C20 (immobile)**	**Order Parameter**	**Immobile Fraction**
Without DNA			
**1:0:0**	0	0	0.54
**1:0:0.2**	0.5	0.042	0.56
**1:0:0.5**	1	0.086	0.56
**1:0:1**	2	0.18	0.56
**1:0:2**	2	0.18	0.6
**1:0:3**	1.8	0.16	0.6
**1:0:5**	1.8	0.16	0.6
With DNA			
**1:1:0**	0	0	0.54
**1:1:0.2**	0	0	0.41
**1:1:0.5**	0	0	0.42
**1:1:0.7**	0	0	0.43
**1:1:1**	1.5	0.13	0.73
**1:1:1.5**	1.5	0.13	0.73
**1:1:2**	1.8	0.16	0.43
**1:1:2.5**	1.8	0.16	0.43
**1:1:3**	1.8	0.16	0.43
**1:1:4**	1.8	0.16	0.43
**1:1:5**	1.8	0.16	0.43

Both components have g values of g_{xx} = 2.0088, g_{yy} = 2.0058, and g_{zz} = 2.0028. Immobile component has hyperfine values of A_{xx} = 16, A_{yy} = 16, and A_{zz} = 105 MHz. Mobile component has hyperfine values of A_{xx} = 16, A_{yy} = 16, and A_{zz} = 103 MHz. The rotational correlation times of immobile and mobile components are 5.2 ns and 0.51 ns, respectively. The C20 value of mobile component stayed constant at 0 for each simulation. Full width at half maximum Lorentzian line broadening values for components 1 and 2 are [0.0025 0.3] mT and [0.018 0.09] mT, respectively. The betaD parameter is 15 degrees for both components. The following experimental parameters were used for the simulations: The frequency of experiment was about 9.88 MHz, and the center field was 3520 G with 120 G sweep width. The modulation amplitude was 1 G, and the temperature of the experiments was 291 K.

Table S2: Parameters derived from MOMD simulations for CueR_A16R1

	**Immobile T_{corr} (ns)**	**Immobile Fraction**	**Mobile T_{corr} (ns)**
Without DNA			
**1:0:0**	3.4	0.51	0.48
**1:0:0.2**	1.5	0.51	0.48
**1:0:0.5**	1.5	0.51	0.15
**1:0:1**	1.5	0.51	0.15
**1:0:1.5**	1.3	0.51	0.15
**1:0:2**	1.0	0.51	0.15
**1:0:3**	1.1	0.51	0.15
With DNA			
**1:1:0**	3.4	0.51	0.48
**1:1:0.2**	2.5	0.51	0.60
**1:1:0.5**	2.4	0.51	0.60
**1:1:0.7**	2.2	0.51	0.60
**1:1:1**	2.0	0.51	0.48
**1:1:1.5**	2.3	0.51	0.48
**1:1:2**	2.0	0.51	0.48
**1:1:2.5**	1.7	0.51	0.48
**1:1:3**	1.3	0.51	0.48
**1:1:4**	1.3	0.51	0.48

Both components have g values of g_{xx} = 2.0088, g_{yy} = 2.0058, and g_{zz} = 2.0028. Immobile component has hyperfine values of A_{xx} = 16, A_{yy} = 16, and A_{zz} = 105 MHz. Mobile component has hyperfine values of A_{xx} = 16, A_{yy} = 16, and A_{zz} = 103 MHz. The C20 values of both components stayed constant at 0 for each simulation. Full width at half maximum Lorentzian line broadening values for immobile and mobile components are [0.0044 0.068] mT and [0.036 0.149] mT, respectively. The betaD parameter is 15 degrees for both components. The following experimental parameters were used for the simulations: The frequency of experiment was about 9.88 MHz, and the center field was 3520 G with 120 G sweep width. The modulation amplitude was 1 G, and the temperature of the experiments was 291 K.

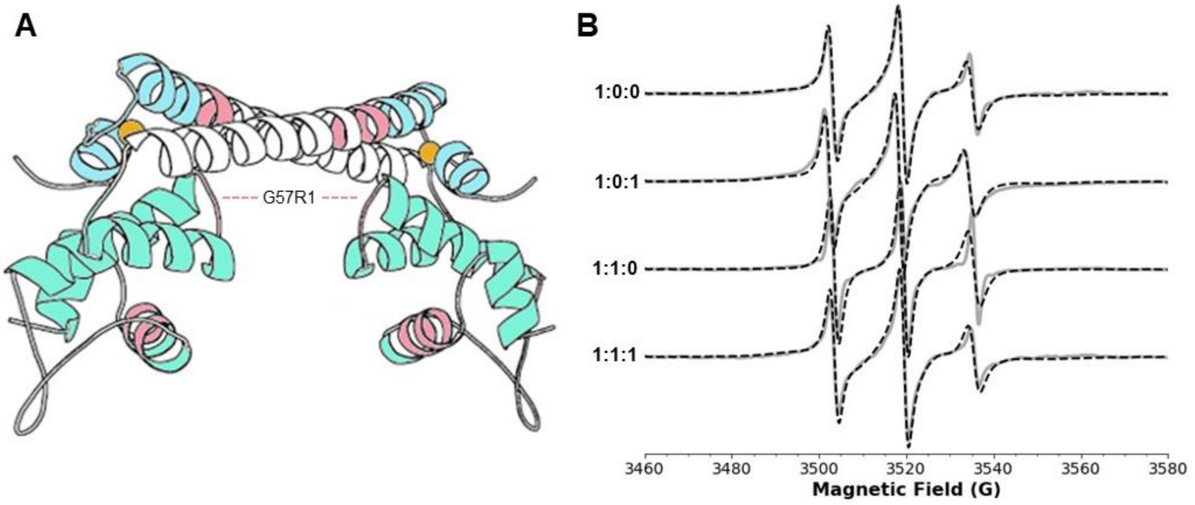

Figure S5: RT CW-EPR spectra (solid gray lines) for CueR_G57R1 at various states. Dashed black lines represent the fitted spectra, fitted parameters are listed in Table S3.

Table S3: Parameters derived from MOMD simulations for CueR_G57R1

	**Component 1 Fraction**
**1:0:0**	0.23
**1:0:1**	0.36
**1:1:0**	0.01
**1:1:1**	0.26

Both components have g values of g_{xx} = 2.0088, g_{yy} = 2.0058, and g_{zz} = 2.0028. Component 1 has hyperfine values of A_{xx} = 16, A_{yy} = 16, and A_{zz} = 105 MHz. Component 2 has hyperfine values of A_{xx} = 16, A_{yy} = 16, and A_{zz} = 103 MHz. The rotational correlation times of components 1 and 2 are 4.6 ns and 0.50 ns, respectively. The C20 values of both components stayed constant at 0 for each simulation. Full width at half maximum Lorentzian line broadening values for components 1 and 2 are [0.0044 0.068] mT and [0.036 0.149] mT, respectively. The betaD parameter is 15 degrees for both components. The following experimental parameters were used for the simulations: The frequency of experiment was about 9.88 MHz, and the center field was 3520 G with 120 G sweep width. The modulation amplitude was 1 G, and the temperature of the experiments was 291 K.

Error in order parameter, rotational correlation time, and $\frac{H_+}{H_0}$

The error in the ordering coefficient was determined by manually decreasing and increasing the coefficient until the RMSD changed. The ordering coefficient was converted into order parameter, and the error in order parameter was determined by propagation of the following equation [1]:

$$S= \int P(\theta)\, Y_{20}(\theta)\, \sin(\theta)\, d\theta$$

where $P(\theta)=e^{-U/kT}$, $U= -(k_BT)(C_{20})(Y_{20})$, $Y_{20}=(\frac{\sqrt{\frac{5}{\pi}}}{4})(3\cos^2\theta-1)$ and θ ranged from 0 to $\frac{\pi}{2}$.

To determine the error in τ_{corr}, the RMSD was calculated as a function of $\log(\tau_{corr})$. The error in $\log(\tau_{corr})$ was set to equal the range in which the RMSD was minimized. The error was converted into an error in τ_{corr}. See the figure below for an example.

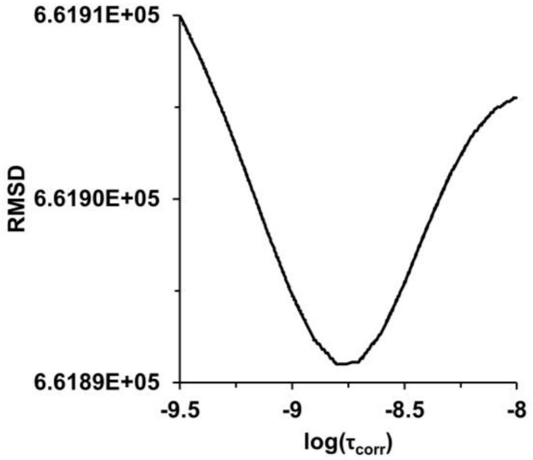

The error in $\frac{H_+}{H_0}$ was propagated using the noise calculated from the experimental baseline at 3470 G to 3472 G.

$$\text{Standard deviation in } \frac{H_+}{H_0} = \sqrt{\frac{\partial H_+}{\partial H_0}^2 \text{noise}^2 + \frac{\partial H_0}{\partial H_+}^2 \text{noise}^2}$$

1. Meirovitch, E., Nayeem, A., and Freed, J. H. (1984) Analysis of Protein Lipid Interactions Based on Model Simulations of Electron-Spin Resonance-Spectra, *J Phys Chem-Us 88*, 3454-3465.
